# Resource selection by a megaomnivore in a marine foraging habitat

**DOI:** 10.1002/ece3.70132

**Published:** 2024-11-03

**Authors:** Andrew S. Maurer, Tomo Eguchi, Garrett E. Lemons, Robin A. LeRoux, Erin L. LaCasella, Calandra N. Turner Tomaszewicz, Megan E. Hanna, Jessica Curran, Bryant Chesney, Sheila V. Madrak, Jeffrey A. Seminoff

**Affiliations:** ^1^ National Research Council Washington District of Columbia USA; ^2^ NOAA Southwest Fisheries Science Center La Jolla California USA; ^3^ United States Navy, Naval Facilities Engineering Command Southwest San Diego California USA; ^4^ United States Navy, Navy Region Southwest San Diego California USA; ^5^ NOAA West Coast Regional Office Long Beach California USA; ^6^ San Diego Miramar College San Diego California USA

**Keywords:** *Chelonia mydas*, Fastloc, resource selection function, satellite telemetry, sea turtle, seagrass

## Abstract

Habitat‐based approaches to animal conservation are bolstered by an understanding of resource selection, that is, use of resources (i.e., habitat features) relative to their availability in the environment. Quantifying resource selection is especially valuable when data characterizing animal space use are limited, as is often the case with mobile and/or cryptic species. Documenting associations with habitat features can better inform management in space in time, while also revealing key insight into movement ecology and behavior. Here, we evaluate resource selection by a megaomnivore whose highly mobile nature within marine habitats has resulted in an incomplete understanding of drivers of space use. We used satellite telemetry to track 29 green turtles (*Chelonia mydas*) from an eastern Pacific foraging aggregation in San Diego Bay, California, USA during 2013–2023. Tracking produced 5023 Fastloc‐GPS points which we used to model selection for local environmental resources relative to their availability. We employed logistic models to evaluate associations with seagrass, bathymetry, and water temperatures, implementing a framework that additionally allowed us to explore the roles of season, diel period, and turtle body size. Our methods demonstrate an approach for down‐weighting observations according to assumed telemetry error and autocorrelation. Results from fine‐scale resource selection models provide evidence that green turtles in San Diego Bay select for eelgrass meadows (*Zostera marina*), particularly during the warmest months of the year, but the strength of this selection changes from day to night. We additionally found day–night shifts in depth and temperature selection that changed with turtle body size and season. We discuss these findings in the context of diel patterns in resting and foraging behavior in addition to seasonal changes in thermally sensitive metabolic rates. Our study documents resource associations and provides quantitative information for the management of sea turtle foraging populations and their habitats. We offer key insight into habitat use by green turtles in the eastern Pacific at a pivotal time when multiple indicators point to population growth and expansion within the region.

## Graphical abstract

We used satellite telemetry data for 29 green turtles (*Chelonia mydas*) in San Diego Bay, California, USA to analyze selection for seagrass, bathymetry, and water temperatures relative to their local availability. We found that green turtles in San Diego Bay select for eelgrass meadows (*Zostera marina*) and certain depths and water temperatures, but patterns were contingent on diel period, turtle body size, and season. We present quantitative resource associations representing actionable information for management at a pivotal time when multiple indicators point to green turtle population growth and expansion within the eastern North Pacific region.

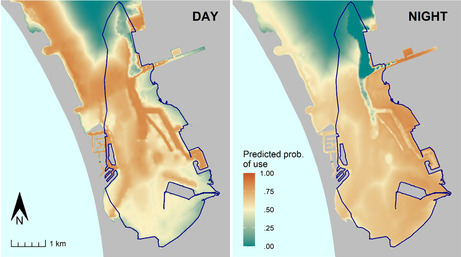

## 1 INTRODUCTION

Conserving animal populations requires an understanding of patterns in space use—that is, *where* individuals occur and, ideally, *why* they occur there (i.e., biotic and abiotic drivers within habitats). Understanding *why* can be crucial, as animal space use patterns may be complex and dynamic, yet often associate with habitat features in predictable ways (Bastille‐Rousseau & Wittemyer, 2021; Manly et al., 2002; Nathan et al., 2022). Indeed, describing habitat associations can greatly aid and simplify animal conservation, a reality borne out in the fact that conservation is often practiced foremost via management of habitat (Nielsen et al., 2006; Watson et al., 2014). Thus, there is a clear need for integrative approaches for associating patterns in animal movement and occurrence with environmental data. Moreover, in certain contexts, the value of quantifying habitat associations is amplified. For example, with many rare, mobile, and/or cryptic species, location/occurrence data are sparse due to the logistical complexity of observing and sampling individuals. In such contexts, capitalizing on limited data to quantify spatiotemporal patterns in habitat and resource use can provide essential information serving as the basis for habitat‐based conservation (e.g., Dellinger et al., 2020; Spencer et al., 2011).

Herein, we explore the use of environmental resources relative to their availability (i.e., resource selection) in sea turtles, a group of marine megafauna whose highly migratory and often solitary life history strategies make them relatively difficult to study. Researchers have characterized sea turtle space use through methods such as boat‐based surveys (e.g., Eguchi et al., 2007), in‐water capture–recapture (Siegwalt et al., 2020), photo identification (Neves‐Ferreira et al., 2023), and biotelemetry (Griffin et al., 2020; Pillans et al., 2022). Satellite telemetry represents perhaps the most informative tool, providing location data with high temporal resolution over relatively long durations (Hays & Hawkes, 2018). These approaches have built substantial knowledge about the locations of foraging and breeding home ranges as well as the migratory corridors that connect them. However, we know comparatively little about fine‐scale space use patterns, that is, how turtles move *within* a home range, and we know even less about what *drives* those movements.

Analyses of resource selection represent a useful avenue to elucidate fine‐scale patterns in space use, particularly as they relate to environmental resources (Manly et al., 2002). Spatial resource selection functions (RSFs) model use of environmental variables (i.e., resources) relative availability, with “selection” inferred when use is disproportionately higher than availability. RSFs can be applied at different spatial scales according to the biological process of interest (Boyce, 2006). At a relatively broad scale, conservation practitioners would benefit from understanding how habitat features relate to where animals occur on the landscape, that is, the locations of movement corridors and/or home ranges (e.g., van Zinnicq Bergmann et al., 2022). Information at a finer scale, such as within a home range, can reveal drivers of spatiotemporally dynamic movement patterns that may underlie local management decisions (e.g., Suraci et al., 2020). Despite the utility of RSFs for exploring multi‐scale patterns in space use, they have rarely been applied to sea turtles. This fact is likely related to the difficulties associated with obtaining quality fine‐resolution habitat data, plus significant modeling obstacles to RSFs implemented with marine telemetry data (i.e., spatial error and autocorrelation). We offer new approaches for addressing such obstacles (see methods section), allowing us to present unique inference into fine‐scale patterns in sea turtle movement.

We evaluate resource selection by green turtles (*Chelonia mydas*), an imperiled species that occurs within tropical and subtropical habitats throughout the world (Seminoff, 2023; Seminoff et al., 2015). We focus on space use within foraging home ranges where, like all sea turtle species, green turtles spend the vast majority of their time (e.g., Webster et al., 2022). Individuals may transition through a series of disparate foraging home ranges before reaching adulthood, at which point they tend to establish fidelity to a single foraging area and commence periodic migrations to reproductive habitats (Jones & Seminoff, 2013; Shimada et al., 2020). Green turtle home ranges are typically distributed in coastal, neritic areas (Jones & Seminoff, 2013), and movements within these areas may be shaped by a myriad of factors. Primary abiotic drivers include bathymetry and water temperatures (Madrak et al., 2022), in addition to currents associated with tides or channelization (Brooks et al., 2009). Biotic factors range from predation risk (e.g., Heithaus et al., 2008) and intraspecific interactions (Mullaney et al., 2024) to, perhaps most importantly, the distribution of food resources. Green turtles had a historical reputation as an herbivorous seagrass and algae consumer, with seminal links to seagrass habitats (Bjorndal, 1980; Frazier, 1971). However, an expanding global knowledgebase on diet composition suggests that omnivory is widespread (Burkholder et al., 2011; Esteban et al., 2020; Gama et al., 2021; Santos et al., 2015; Seminoff et al., 2021), raising important questions about habitat use and resource selection at local scales, for example, are seagrass meadows necessarily a foundational resource?

We address questions of resource selection for a population of green turtles within the Eastern Pacific in San Diego Bay (hereafter SDB), California, USA. SDB hosts one of the largest seagrass expanses on USA's west coast (primarily eelgrass *Zostera marina*; Merkel & Associates, 2020), but clearly demonstrated regional and local diet plasticity reinforce uncertainty regarding the importance of eelgrass to habitat selection and space use (Clyde‐Brockway et al., 2022; Esteban et al., 2020; Lemons et al., 2011; López‐Mendilaharsu et al., 2005, 2008; Quiñones et al., 2022; Seminoff et al., 2002, 2021; Turner Tomaszewicz et al., 2018). We used GPS data collected over 11 years to explore whether green turtles exhibited selection for eelgrass habitats along with key abiotic variables, herein presented as “resources”: depth and water temperature. Our RSF framework additionally tested hypotheses that behavior would vary according to turtle body size and time period (i.e., day vs. night and season). We anticipated that behavior would vary with body size given previously documented physiological and diet variation among green turtle age classes (Jones & Seminoff, 2013). Expectations for diel and seasonal changes in resource selection were based on links among photic conditions, water temperatures, and behavior (e.g., Christiansen et al., 2016). By quantifying fine‐scale habitat associations, our findings can help produce better‐informed management strategies and open inferences into new aspects of sea turtle movement ecology.

## 2 METHODS

### 2.1 Data collection

#### 2.1.1 Study system

Green turtle capture and satellite telemetry took place in SDB. The bay is ~45 km^2^ in area and serves as an important estuarine habitat for regional marine flora and fauna, including several protected species (Allen et al., 2023; Eguchi et al., 2020; Merkel & Associates, 2020). Simultaneously, the bay is among the most urbanized estuaries on the US West Coast. Human development nearly completely encircles the coastline, including multiple US Naval bases, a commercial port, and several marinas. Consequently, the bay is subjected to significant human contamination (Komoroske et al., 2011) and dense vessel traffic from Navy, commercial, and recreational boats.

SDB has an elongated shape with an extended north–south axis. Previous studies divided the bay into four ecoregions along this axis (e.g., Allen et al., 2023). The northernmost region adjacent to the bay's mouth has deeper water with greater tidal exchange, whereas the southernmost region is characterized by relatively shallow, warm habitats with less exchange and minimal dredging. It is in this southernmost region, hereafter South Bay, that green turtle foraging activity has long been concentrated (Eguchi et al., 2020; MacDonald et al., 2012; Stinson, 1984). The most recent abundance estimates placed the resident foraging population between approximately 30 and 60 individuals (over 2001–2009; Eguchi et al., 2010), but anecdotal evidence, including rates of new captures, suggests marked increases in recent years. The foraging population comprises a mix of size classes, with natal nesting beaches largely located further south in Mexico (Dutton et al., 2018).

Several factors likely contribute to the concentration of green turtles within South Bay. Shallow depths lead to reduced boat traffic and yield more suitable substrate for submerged aquatic vegetation and associated consumers. Indeed, the bulk of SDB's eelgrass occurs in South Bay (Merkel & Associates, 2020). Turtles may also be drawn to the warmer waters. Previously, high green turtle densities in South Bay appeared to be associated with artificial warming by effluent from an adjacent powerplant (Turner‐Tomaszewicz & Seminoff, 2012). Despite the decommissioning of the plant in 2010, South Bay's waters naturally remain warm relative to the rest of San Diego Bay (Madrak et al., 2016), and turtles have maintained residency in the area without the warm effluent (Eguchi et al., 2020). Herein, we focus solely on green turtle movement after the closure of the powerplant; previous studies have compared behavior before and after plant closure (Eguchi et al., 2020; Madrak et al., 2016, 2022).

#### 2.1.2 Turtle capture and satellite transmitter deployment

The green turtles tracked in this study were captured as part of annual mark–recapture monitoring in South Bay (capture site located at ~32.613°, −117.097°). Capture methodology has been described previously (e.g., Eguchi et al., 2010, 2020). Briefly, we used 50‐ to 100‐m long and 8‐m deep entanglement nets (40‐cm knot‐to‐knot mesh size) that were set within 100 m of shore and checked every 30 min at minimum. Captured turtles were hauled aboard a 4‐ to 6‐m Boston Whaler skiff with outboard motor and transported to a shore‐based work station where a research team took measurements, collected tissue samples, and attached satellite transmitters. The sampling process included assigning putative sex for sexually mature turtles and measuring straight carapace length with calipers (from the nuchal notch to the end of the posterior marginal scutes).

**FIGURE 1:**
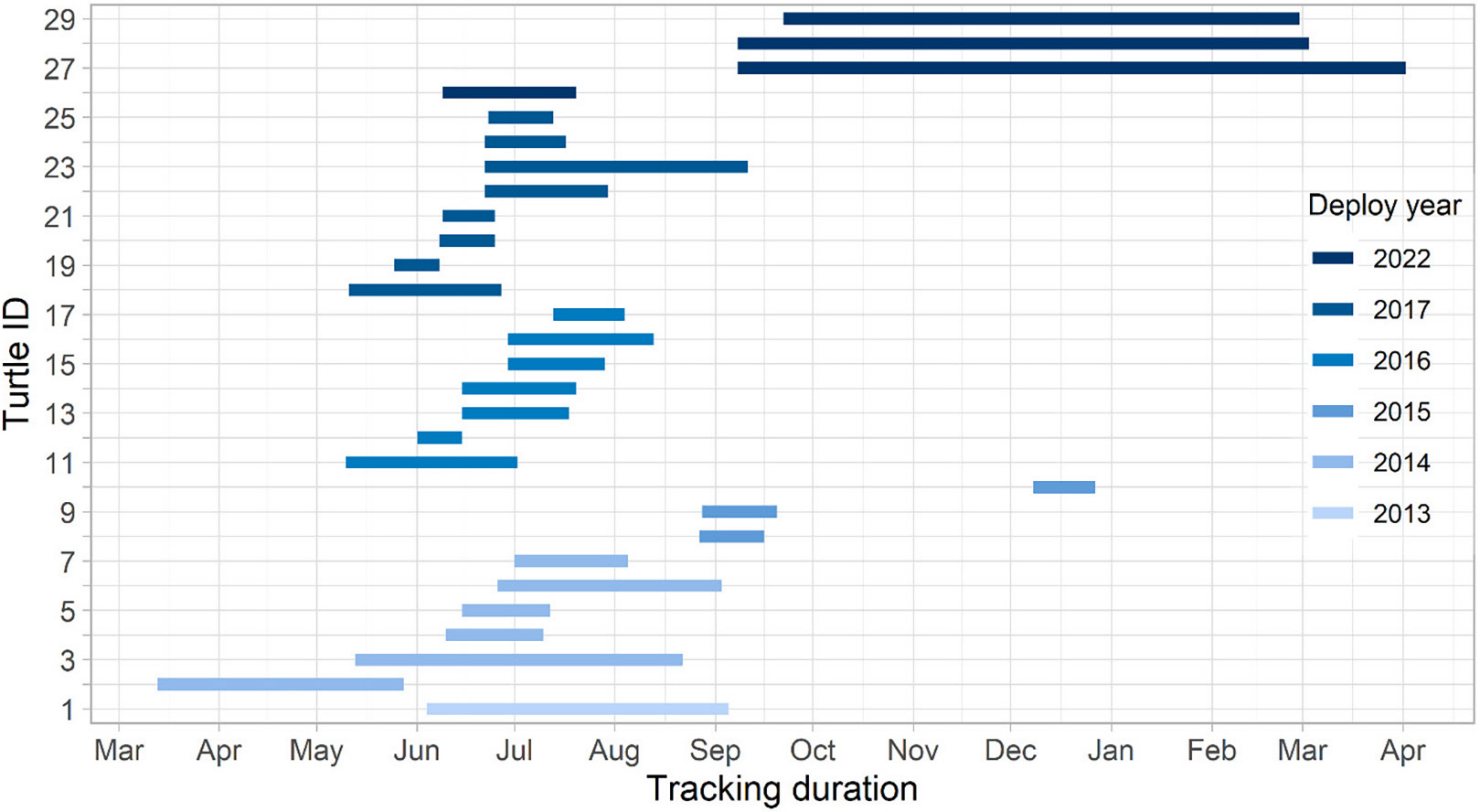
Satellite transmitter observation periods for green turtles (*Chelonia mydas*) tracked in San Diego Bay, California, over 2013–2022. Durations are based on GPS data after user filtering.

We deployed Argos‐linked Fastloc‐GPS transmitters to track turtle movements within San Diego Bay during 2013–2023. This period includes new data in addition to several transmitter deployments previously summarized (through 2016; Eguchi et al., 2020). Deployments typically began during the local warm season (April–August), with some exceptions (Table 1; Figure 1). Wildlife Computers MK10 transmitters were attached as described by Eguchi et al. (2020) following methods outlined by Jones et al. (2011). For each attachment, we used quick‐setting marine epoxy to anchor transmitters to the highest spot on the carapace with the antenna oriented forward.

**TABLE 1:** Demographics and satellite transmitter deployment summaries for green turtles (*Chelonia mydas*) sampled in San Diego Bay, California over 2013–2022.

Individual	Sex	SCL (cm)	Tracking start	Duration (days)	*N*	N_EFF_
1	F	100.5	6/4/2013	92.6	81	81
2	F	109.3	3/13/2014	75.5	189	189
3	F	95.0	5/13/2014	100.7	201	201
4	U	65.8	6/10/2014	29.2	43	43
5	M	102.6	6/15/2014	27.9	7	7
6	F	100.8	6/26/2014	68.7	216	216
7	F	99.0	7/1/2014	34.3	113	113
8	U	75.2	8/27/2015	19.3	66	66
9	F	102.5	8/28/2015	23.3	69	69
10	F	94.8	12/8/2015	18.9	85	30.5
11	F	101.3	5/10/2016	52.9	42	42
12	U	69.2	6/1/2016	13.3	52	39.7
13	U	80.4	6/15/2016	33.1	33	33
14	U	62.3	6/15/2016	34.4	140	87.6
15	U	65.4	6/29/2016	29.5	145	97.7
16	U	71.0	6/29/2016	44.8	309	195.0
17	U	71.3	7/13/2016	21.2	221	101.4
18	U	79.7	5/11/2017	47.0	105	82.1
19	U	61.6	5/25/2017	13.3	110	27.9
20	U	75.8	6/8/2017	16.8	112	71.1
21	U	83.0	6/9/2017	16.6	6	6
22	U	53.2	6/22/2017	38.1	26	15.8
23	F	90.5	6/22/2017	80.1	241	147.6
24	U	40.0	6/22/2017	24.8	103	58.3
25	M	88.1	6/23/2017	20.2	33	27.7
26	M	66.5	6/9/2022	40.9	134	134
27	F	95.3	9/8/2022	205.9	1039	738.4
28	U	63.3	9/8/2022	175.9	671	571.7
29	F	83.3	9/22/2022	158.8	431	298.9

*Note*: *N* is the number of relocations (i.e., sample size of GPS fixes) and N_EFF_ is the estimated effective sample size after accounting for temporal autocorrelation. A sex of “U” denotes unidentifiable sex.

#### 2.1.3 GPS data filtering

Fastloc‐GPS data were relayed via the Argos satellite system and managed by the Wildlife Computers data portal. Spatial accuracy for satellite fixes can be affected by time at the surface, the number of satellites overhead, and their positioning. Information on accuracy is represented by a “residual value” and a count of satellites per GPS location (Dujon et al., 2014). We downloaded and applied several filters to the GPS data. First, we discarded all relocations with a residual value ≥35, a threshold indicating potentially high spatial inaccuracy (Dujon et al., 2014). Next, to focus on just foraging behavior, we excluded turtles that made longer‐range movements outside of SDB. Additionally, we removed any relocations that erroneously fell outside of the bay (i.e., over land or over the ocean to the west). We note that GPS data reflect surfacing locations and are therefore not a complete record of movement. Nonetheless, we assumed that high‐volume GPS data provided an unbiased representation of movement, and we checked for temporal bias by examining the frequency of relocations within each hour of the day.

#### 2.1.4 Spatial habitat data

**FIGURE 2:**
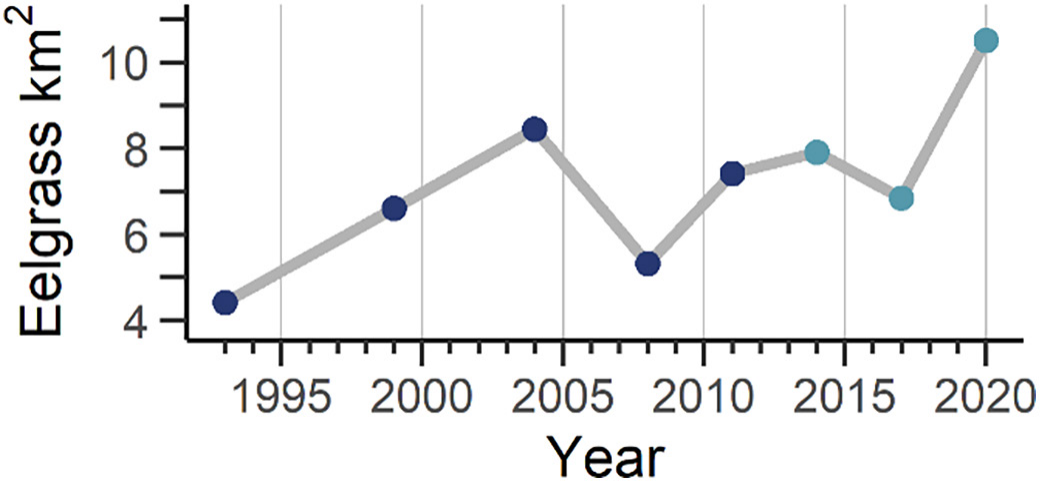
Eelgrass (*Zostera marina*) coverages through time in San Diego Bay, California. Areas were calculated based on side‐scan sonar surveys of the full extent of the bay conducted by the US Navy and contracted associates, as reported by Merkel & Associates (2020). Light blue dots denote years of focus for our study tracking green turtle (*Chelonia mydas*) movement in San Diego Bay.

**FIGURE 3:**
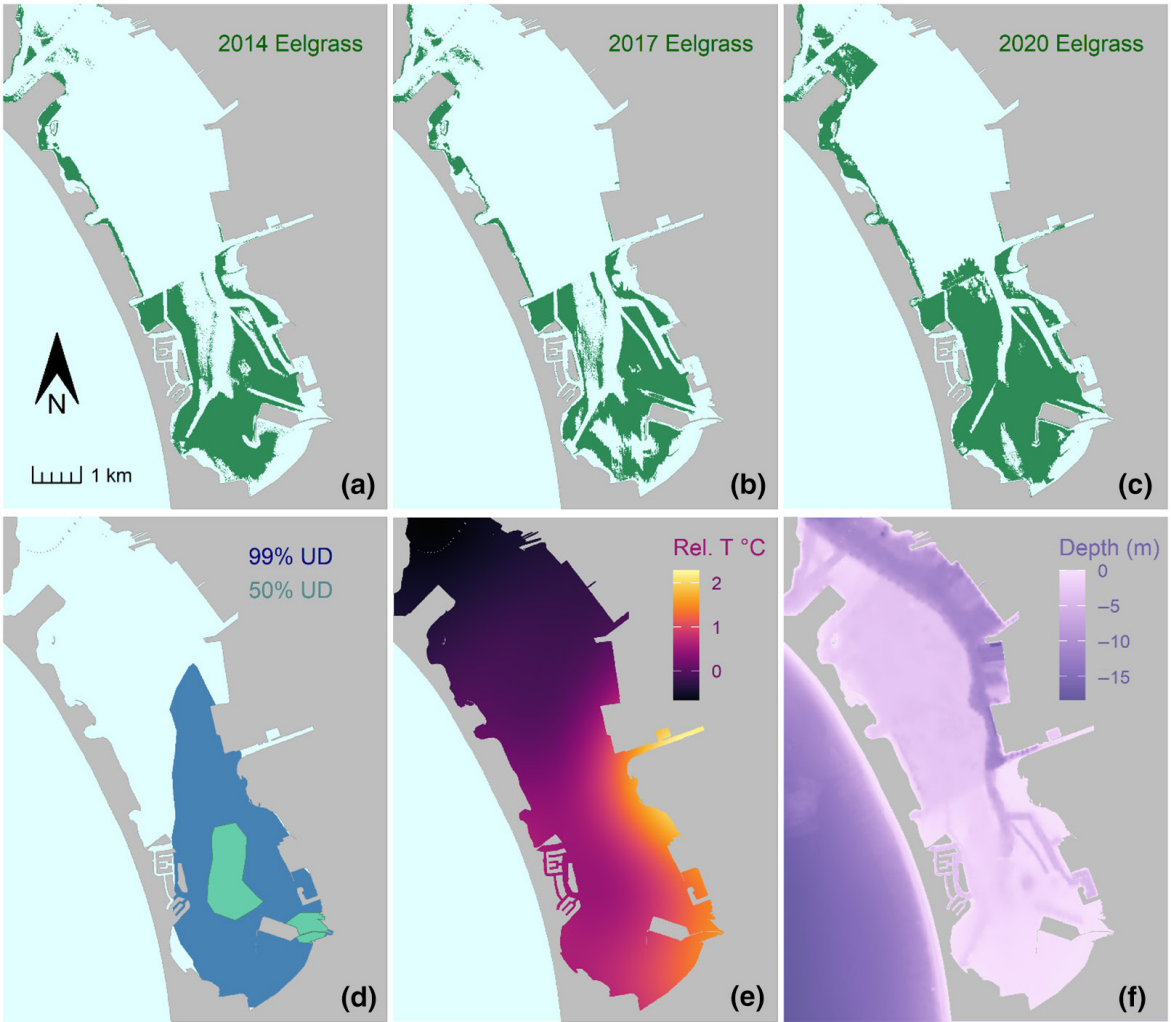
Green turtle (*Chelonia mydas*) habitat information in south San Diego Bay, California, USA. Panels illustrate information as follows. (a–c) Eelgrass (*Zostera marina*) coverages in 2014, 2017, and 2020 documented by Merkel & Associates (2020). (d) The 99% and 50% kernel utilization distributions (UDs) computed from all GPS points collected for 29 turtles (after GPS data filtering). (e) Mean relative water temperatures interpolated from two data sources (see methods for details). (f) Water depth, that is, bathymetry (OCM Partners, 2016).

Eelgrass – The spatial distribution of eelgrass within San Diego Bay has been mapped via sonar at 3‐ to 6‐year intervals starting in 1993 (Merkel & Associates, 2020). These surveys were conducted by the US Navy, and more recently contracted to Merkel & Associates, Inc., through cooperation by Naval Facilities Engineering Command Southwest and the Unified Port of San Diego. We summarize all available eelgrass surveys 1993–2020, spanning multiple improvements in survey technology (Figure 2), but our analyses focus on just the last three surveys (2014, 2017, 2020; Figure 3a–c) that all employed the same method featuring interferometric side‐scan sonar. We obtained polygon shapefiles reflecting eelgrass coverages and converted them to polylines (i.e., polygon edges) to enable us to calculate the distance from a given location to the nearest eelgrass edge, with positive distances reflecting locations outside of eelgrass polygons and negative distances assigned to locations overlapping eelgrass habitat. We underscore that eelgrass habitats are dynamic—changes in distribution, biomass, and community composition can play out according to seasonal cycles or result from perturbation (Hasegawa et al., 2007; Munsch et al., 2023). Although we suggest that access to triennial, high‐resolution eelgrass coverages is rare and presented a unique opportunity to assess habitat use, we acknowledge an inability to account for changes in biomass or composition between surveys. Further, we assumed that inaccuracy resulting from changes in eelgrass edges between surveys and the time of turtle tracking did not confound inference. Specific model parameter estimates should accordingly be interpreted with caution, yet broader inferences (e.g., whether model effects were highly significant) are likely robust to inter‐survey eelgrass dynamism. We elaborate on these ideas below in the context of modeling methods. Eelgrass layers used the California Albers projection (1‐m resolution); all other layers were designated or reprojected to match.

Bathymetry – We obtained bathymetry data from a publicly available USGS topobathymetric model (1‐m spatial resolution; OCM Partners, 2016). This raster integrates 49 data sources and was created specifically for Southern California. Herein, we use the term “depth” to refer to bathymetric depth. We emphasize that this represents the depth of the habitat used by turtles (i.e., distance from sea level to floor at a given location), not the position of turtles within the water column.

Water temperatures – We used data from two sources to estimate the spatial distribution of mean sea surface temperatures in SDB. We integrate over a time series of measurements to present mean water temperatures at given locations relative to the overall SDB mean (i.e., delta values), in effect creating a static map of warmer versus colder areas in the bay, which was appropriate for our spatial analysis as compared to focusing on absolute temperatures that can vary substantially on daily timescales (temporal effects are explored via model interaction terms; see section 2.2). We related repeated surface temperature measurements (at a depth of 1 m) across 113 stations within a 500‐m grid (Madrak et al., 2016) to data from the local NOAA buoy SDBC1 (32.714°, −117.174°) to derive a mean temperature for each grid sampling station. After converting these to relative delta values, we interpolated a continuous raster surface for SDB via ordinary kriging. More details on data integration and interpolation are provided in Appendix 1. We assumed that relative differences in sea surface temperature within SDB remained consistent during the study period due to consistent hydrological and bathymetric conditions. We acknowledge that a two‐dimensional treatment of water temperatures ignores three‐dimensional variation, and future treatments would benefit from more information on potential vertical stratification and thermoclines. Given data available to us, this was an effective way to incorporate a key, temporally dynamic environmental parameter in a spatial resource selection model.

### 2.2 Data analysis

#### 2.2.1 Resource selection functions: Use versus availability

Resource selection functions comprise a broad set of models that provide inference into the probability of an area or resource being used by an animal (Manly et al., 2002). We implemented a commonly applied class of spatial RSF in which habitat parameters at “used” locations (observed via telemetry) are compared to artificially generated locations (i.e., pseudo‐absences) within an area deemed “available” to the animal in question. In essence, this approach determines whether an animal's observed movements suggest it is using certain resources at rates disproportionate to what is available in the surrounding environment.

Inferences derived from RSFs are scale‐dependent. For example, an environmental resource that appears unimportant to an animal when considering a small spatial extent (e.g., within which daily movement patterns are shaped) might be more influential over a broader extent (e.g., over which the locations of home ranges are determined; Boyce, 2006). In this vein, RSF inferences are contingent on the area designated as available. We used the observed home range as our extent of inference, a commonly applied approach (Manly et al., 2002). We pooled GPS locations across individual turtles to estimate a joint‐kernel utilization distribution (UD) with the adehabitatHR package in R (Calenge, 2006); we used the default extent parameter and a bandwidth selected via the ad hoc method for a bivariate normal kernel, then masked any land from the resulting polygon. We opted to use the 99% UD contour from all filtered GPS points to represent a conservatively large available habitat and pooled all locations, as opposed to partitioning by individual, as our goal in this context was to generate a polygon capturing the overall footprint of movement in SDB.

To prepare for modeling with GPS locations representing *use*, we first generated sets of point locations (i.e., pseudo‐absences) representing *availability*. All intended models included terms unique to individual turtles, and thus we grouped GPS locations and pseudo‐absences by individual. Our questions regarding temporal differences necessitated further day–night and seasonal subgrouping; thus, the ultimate subgrouping was individual × day–night × season. We delineated day versus night using the timing of sunrise and sunset identified with the StreamMetabolism package in R (Sefick, 2016), and we split data into a warm season (Apr–Nov) and cold season (Dec–Mar) based on monthly mean water temperatures recorded by NOAA buoy SDBC1 (see Appendix 2). We iteratively generated sets of pseudo‐absences to match each ultimate subgrouping of GPS locations, with pseudo‐absences organized into a regular grid within the polygon designating availability. This approach to pseudo‐absences serves to point sample the available area without respect to the location of GPS locations. For example, if a subgrouping (individual × day–night × season) contained 20 GPS locations, we generated 20 pseudo‐absences spaced regularly in a grid within the available polygon. Minor differences between the number of GPS locations and pseudo‐absences occasionally resulted from constraints associated with regular grid spacing within an irregular polygon.

We annotated habitat values from spatial data products to each GPS location and pseudo‐absence, recording the distance to the nearest eelgrass edge, bathymetric depth, and relative water temperature value. Distance to eelgrass edge was calculated based on the eelgrass survey closest in time to that individual's transmitter deployment year; this represented a difference of ≤1 year in all cases except when 2022–2023 GPS points were related to the 2020 survey. Our anecdotal observations in South Bay suggest that conditions did not change appreciably between 2020 and early 2023. Minimal time differences between surveys and turtle tracking helped to minimize inaccurate representation of the eelgrass edge at the time of tracking. Raw GPS data, accompanying turtle demographic information, and annotated GPS and pseudo‐absence locations are provided in the Supporting Information (Data [Link], [Link], [Link]).

#### 2.2.2 Accounting for telemetry error

Fastloc‐GPS technology emphasizes generating location fixes quickly. It was designed this way because transmitters can uplink to satellites only when antennae break the ocean surface, and surfacing episodes may be brief. This ingenuity comes at the cost of accuracy, as Fastloc is more prone to error than conventional, terrestrial GPS applications. There are two Fastloc‐GPS data parameters that indicate relative accuracy for a location—a residual value (mentioned above) and number of satellites (Dujon et al., 2014). After filtering out fixes with problematically high residual values, we then derived a weight term for subsequent modeling where GPS fixes with fewer satellites were down‐weighted. We built the weight term using data provided by Dujon et al. (2014), who conducted experimental trials to associate the number of satellites with real metric error for 45,157 Fastloc locations. We used their raw data, first discarding any error values greater than the 95th quantile (error on that scale was not present in our data) and then calculating a mean error for each number of satellites. We took the multiplicative inverse of these means and, for interpretive ease, rescaled the resulting values so that the largest weight was 1 (Table 2).

**TABLE 2:** Model weights based on the number of satellites contributing to each Fastloc‐GPS fix, derived from the results of Dujon et al. (2014).

Satellites	Model weight
4	0.28
5	0.31
6	0.49
7	0.68
8	0.83
9	1
NA	0.28

*Note*: Computer‐generated pseudo‐absences are denoted by NA.

All computer‐generated pseudo‐absences were assigned the weight of the lowest satellite count such that these arbitrarily generated points did not outweigh any observations. This weight term reflecting telemetry error was then combined with a second weight accounting for autocorrelation, described in the subsequent section. Beyond explicitly accounting for telemetry error with a weight term, we also chose to use only continuous versions of spatial predictor variables in RSFs (i.e., as opposed to categorical variables), leaving parameter estimates less sensitive to GPS imprecision. For example, if representing eelgrass as a binary term indicating whether a point is within or outside an eelgrass patch, then 1 m of telemetry error could lead to a 100% change in the eelgrass response term. In contrast, when representing eelgrass in terms of a continuous distance to edge, 1 m of error has a negligible effect on parameter estimates.

#### 2.2.3 Accounting for autocorrelation

Models of resource selection generally assume that observations are independent and identically distributed. Marine applications of satellite telemetry produce surface fixes at irregular time intervals and thus violate this assumption, introducing temporal autocorrelation and potential pseudoreplication. Rather than, for example, thinning data to reduce autocorrelation, we instead derived a second weight term to down‐weight autocorrelated points (Alston et al., 2023; Fleming et al., 2018; Silva et al., 2022).

For each individual, we used the R package ctmm (Calabrese et al., 2016) to first select a best‐fitting continuous‐time movement model and then used that model to estimate and assign weights to each point, where autocorrelated points are down‐weighted (see Appendix 3 for further detail). We also estimated an effective sample size for each individual, hereafter N_EFF_, roughly reflecting the number of times an animal crossed its home range within the observation period (Fleming et al., 2019; Silva et al., 2022). Higher levels of autocorrelation result in proportionally lower N_EFF_ values. We structured the autocorrelation weight term so that each individual's total weight was equal to its sample size (N_EFF_) and was split equally between its GPS locations and pseudo‐absences. The 50% of total weight for an individual's GPS locations was distributed proportionally to the autocorrelation weights estimated for each location, while the remaining 50% of weight was distributed evenly among pseudo‐absences (given seasonal subgrouping, weight for pseudo‐absences was partitioned first according to the proportion of that individual's N_EFF_ associated with each season, then spread evenly within each seasonal subgroup). This overall structuring meant that turtles with more GPS points (a larger N_EFF_) had proportionally greater weight during modeling, but given that a separate model was developed for each season (described section 2.2.4), no turtles had extreme weight (Figure 1).

Finally, we multiplied the two weight terms, one accounting for telemetry error and one for autocorrelation, to arrive at a single term for modeling. We rescaled the resulting, combined weight term so that its sum matched the sum of GPS locations plus pseudo‐absences, because weights in a binomial regression act as a number of trials and affect parameter estimation (herein our response variable, 0 or 1, would be treated as a binomial proportion, with more “trials” creating more weight). The resulting nonzero model weight values ranged from 0.01 (corresponding with high autocorrelation and low accuracy) to 5.2. A total of 39 GPS locations received zero weight due to maximal autocorrelation.

#### 2.2.4 Model fitting, comparison, and predictions

We used logistic models to evaluate green turtle space use as a function of three aforementioned environmental resources: distance to the nearest eelgrass edge, bathymetry, and water temperature. We fit and compared generalized linear models to explore fine‐scale resource selection within the observed home range, including comparison of daytime versus nighttime behavior, comparison of warm‐season versus cold‐season patterns, and an examination of variation by body size. In this RSF modeling framework, nonspatial predictors (day–night, season, and body size) must be included as interactions with spatial covariates, and thus we originally intended to explore complex models with four‐way interactions (i.e., a spatial resource variable interacting with all three nonspatial variables). However, a limited sample size for the cold season (*N* = 5 individuals) inhibited estimation of certain parameters (i.e., resulted in singularities) associated with three‐ and four‐way interactions with season. Thus, we instead divided the dataset by season and developed separate models for the warm and cold seasons. We additionally note that model convergence failures prevented an approach using mixed‐effects models to model individual variation (i.e., with a random intercepts term)—we prioritized the inclusion of a continuous effect for turtle body size to model individual variation, as this was pertinent to research questions framed for the study.

The response variable for all logistic regressions was binary, with GPS locations assigned a 1 and pseudo‐absences a 0. All models included the weight term accounting for telemetry error and autocorrelation. In separate model comparison exercises for the warm season and cold season, we evaluated different combinations of the following spatial predictors: (1) distance to eelgrass edge, also considering a quadratic effect given the possibility of edge effects; (2) water depth, including a quadratic effect given the potential for selection for certain depth bands; and (3) relative water temperature—we chose a priori to not explore a quadratic term here to avoid overparameterization given relationships with bathymetry, a variable for which we had higher‐resolution data. A fourth spatial predictor, distance to the capture site, was excluded from consideration due to collinearity with both bathymetry and water temperature (in both seasonal datasets). Indeed, we examined collinearity in all spatial predictor terms with simple linear regressions, setting a threshold *R* ^2^ value of .49 to determine whether a pair of predictors was too collinear to include both, as suggested by Dormann et al. (2013). We posit that capture bias is mitigated at the spatial scale of this study, as movements within the home range consistently encapsulate a larger area of which the sample site is one part (Eguchi et al., 2020).

We employed similar model comparison exercises to arrive at a single best performing model of resource selection in both the warm and cold season. This entailed a three‐phase approach, where models considered in phases two and three were informed by empirical information from previous phases. While a classic approach to model comparison is to outline all models to be considered a priori, here we intended to explore biologically relevant three‐way interactions, entailing large number of possible model structures. The phased approach allowed us to streamline model comparison within this context.

In phase one, we compared four basic models including just the spatial predictor terms and no interactions. We used results to empirically inform a new set of candidate models for comparison in phase two, in this case including all possible interactions with a single nonspatial variable at a time (i.e., day–night or body size, but not both in a single model). Finally, in phase three, we built out from the best‐performing phase‐two model to then consider more complex models with three‐way interactions. Appendix 4 details all model structures considered, including 25 models for the warm season and 25 for the cold season. We compared models using AICc and selected a single top‐performing model for inference, simplifying interpretation as compared to model averaging when our primary aim was to evaluate variable effects. When selected models contained two‐way interactions with factors or three‐way interactions, we used a Type II analyses of deviance to provide a simplified evaluation of whether model terms significantly improved fit (α = 0.05).

We assessed the explanatory power of models using a variance function‐based coefficient of determination (i.e., pseudo‐*R* ^2^) for generalized linear models (Zhang, 2017). We illustrated interactions and variable effects for the top‐performing model for each season by predicting probability of use with artificially generated input datasets that varied predictors of interest over their ranges and held other predictors constant at their means (the weight term was fixed at its median). When illustrating interactions, we explored both day and night and five different values for body size—in the warm season (*N* = 28 individuals), we used the mean SCL and two increments of one standard deviation in either direction, while in the cold season, we varied SCL evenly between the range of SCLs of the turtles tracked (*N* = 5) during that period. For legibility of figures, we present single, outermost prediction intervals around the five different SCL scenarios (i.e., the maximum interval when considering the five individual 95% intervals combined).

We also made predictions in space; specifically, we used the top logistic model to predict probability of use within South Bay, making a separate prediction for the day versus night, here for a turtle of mean body size (80.9 cm SCL) in just the warm season. We used habitat rasters as input data (bathymetry, sea surface temperature, and the 2020 eelgrass coverage) and generated new rasters reflecting the predicted probability of use, plus uncertainty for predictions, at each raster cell. Although best viewed as a model fit visualization tool, not a form of predicted distribution, these spatial predictions may inform local management.

## 3 RESULTS

### 3.1 Turtle sampling and tracking

After filtering, our final GPS dataset consisted of 5023 locations distributed among 29 turtles, with three putative males, 11 females, and 15 of unknown sex (Table 1). These individuals had a mean ± SD straight carapace length of 80.9 ± 17 cm, ranging 40.0–109.3 cm. GPS‐enabled transmitter deployments took place in 6 years with a mean duration of 53.7 ± 50 days, a minimum of 13.3 days, and maximum of 205.9 days (Figure 1). This included one transmitter deployed in 2022 that was still active at the time of data acquisition in April 2023. The 29 transmitter deployments produced a mean of 173.2 ± 217 GPS locations per individual. After accounting for autocorrelation, this corresponded to a mean estimated effective sample size (N_EFF_) of 130.7 ± 163 locations per turtle.

**FIGURE 4:**
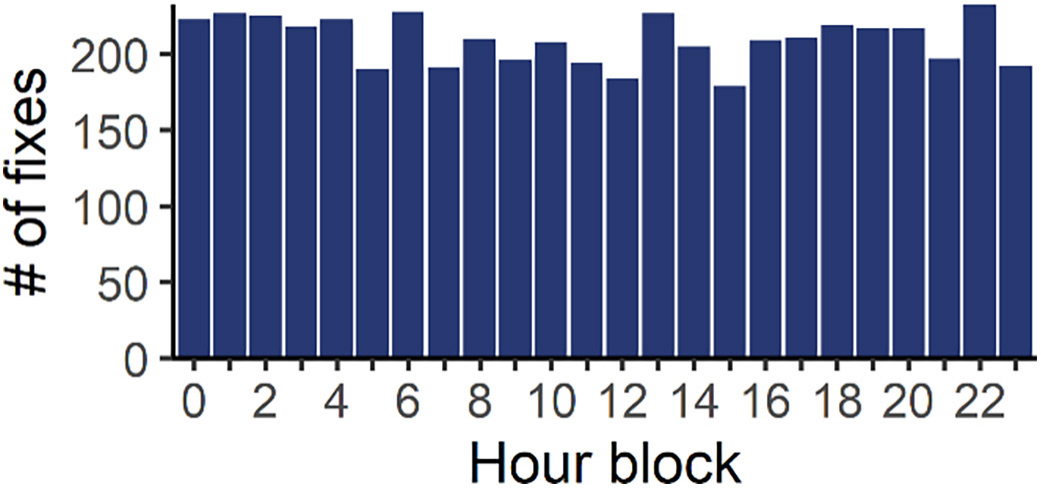
Satellite fix frequency by hour of the day for the combined GPS datasets of 29 green turtles tracked in San Diego Bay, California.

The full GPS dataset was not biased to certain diel periods, with fixes distributed relatively evenly among hours of the day (Figure 4). As delineated by sunrise and sunset, 51% of fixes occurred during the day and 49% at night. The dataset was strongly biased to the warm season, with 85% of locations occurring Apr–Nov. Thus, we had much stronger inference into warm‐season patterns during modeling. Five individuals provided cold‐season data, while 28 provided warm‐season data (four individuals spanned both cold and warm seasons).

**FIGURE A3:**
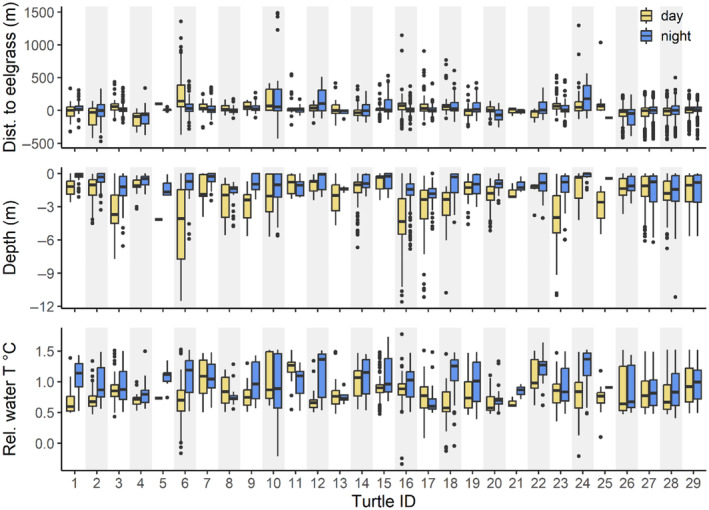
Box and whisker plots illustrating distance to nearest eelgrass edge (*Zostera marina*), bathymetric water depth, and relative water temperatures at GPS locations collected via satellite telemetry for green turtles (*Chelonia mydas*; *N* = 29) in San Diego Bay. Raw, unmodeled data are shown, without seasonal grouping. Colors differentiate daytime versus nighttime behavior. Distance to eelgrass is expressed as a distance to patch edge, with locations inside a patch assigned negative values.

The 99% UD computed using all 5023 GPS points had an area of 12.37 km^2^ and was restricted primarily to South Bay (Figure 3d). We emphasize that this UD encompasses observed “area‐restricted search” behavior only; indeed, several turtles have been documented making migratory or ranging movements through and outside the bay but these individuals were excluded from this analysis. A summary of environmental resource data referenced to raw, unmodeled GPS locations for individual turtles (i.e., without accounting for availability, telemetry error, or autocorrelation) is provided in Appendix 5 (Figure A3).

### 3.2 Resource selection models

We compared 50 total logistic models to evaluate resource selection at the scale of the population home range (i.e., the 99% UD). The warm‐season models were fit using 4274 GPS locations and 4257 pseudo‐absences, while cold‐season models were informed by 749 and 750, respectively. All covariates passed predefined guidelines for collinearity within both seasonal datasets, with a maximum *R* ^2^ of .47 between water temperature and bathymetry in the cold season.

**FIGURE 5:**
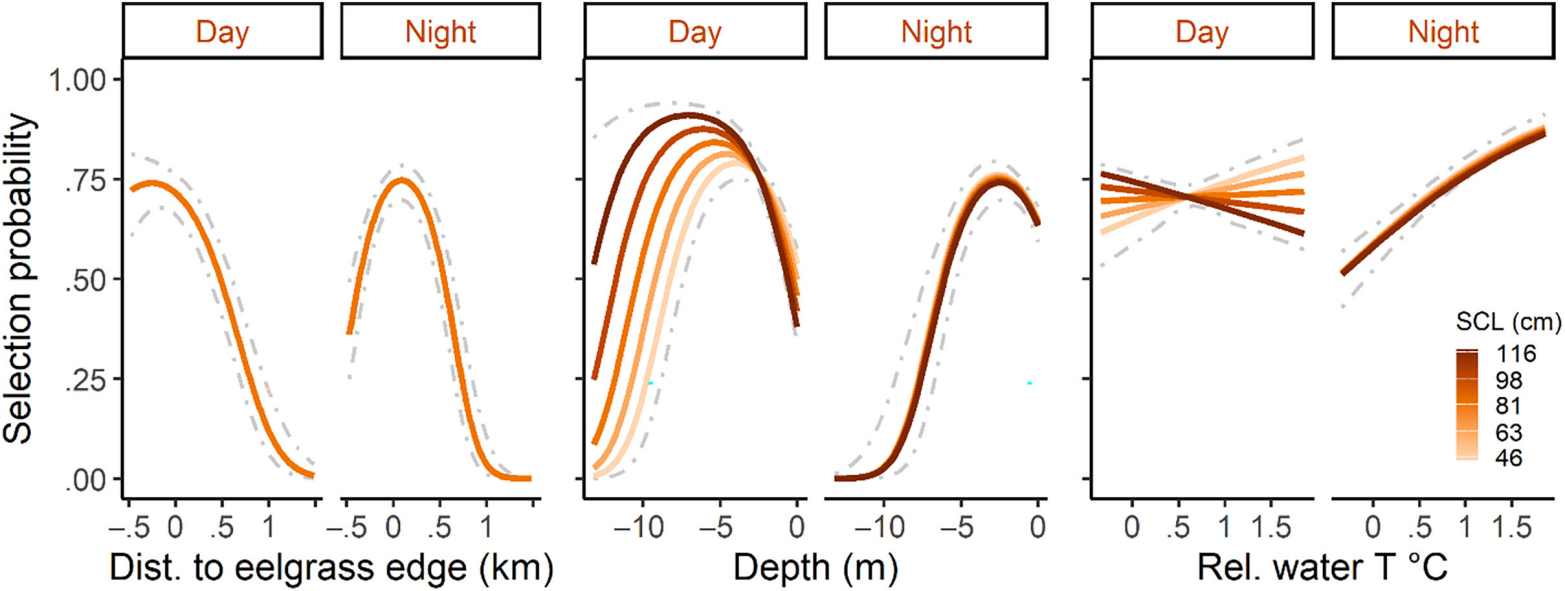
Warm‐season predicted probability of use as a function of distance to the nearest eelgrass (*Zostera marina*) edge, bathymetric water depth, and water temperature for green turtles (*Chelonia mydas*) in San Diego Bay, California. In all cases, significant interactions suggest that habitat associations differed between day versus night periods (left vs. right subpanels, respectively). For depth and water temperature, an additional interaction for depth and temperature suggested further variation among turtles of different sizes (straight carapace length, SCL), depicted here with light to dark hues. We modeled warm‐season resource selection using GPS point locations collected for 28 individuals and designated the joint‐99% utilization distribution as the available area (pseudo‐*R* ^2^ = .087). Locations within seagrass habitats were assigned negative values. Water temperature is presented as a mean relative value. Dashed gray lines display an outermost 95% prediction interval.

**FIGURE 6:**
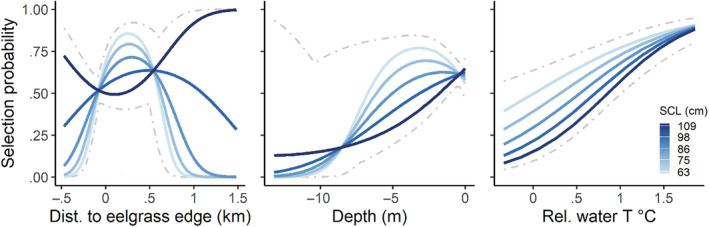
Cold‐season predicted probability of use for green turtles (*Chelonia mydas*) in San Diego Bay, California. Significant interactions suggest that habitat associations varied with turtle body size (straight carapace length, SCL, light to dark hues), but there was no support for day–night interactions in the cold season. Cold‐season resource selection using GPS point locations collected for five individuals (pseudo‐*R* ^2^ = .203). Dashed gray lines display an outermost 95% prediction interval.

**FIGURE 7:**
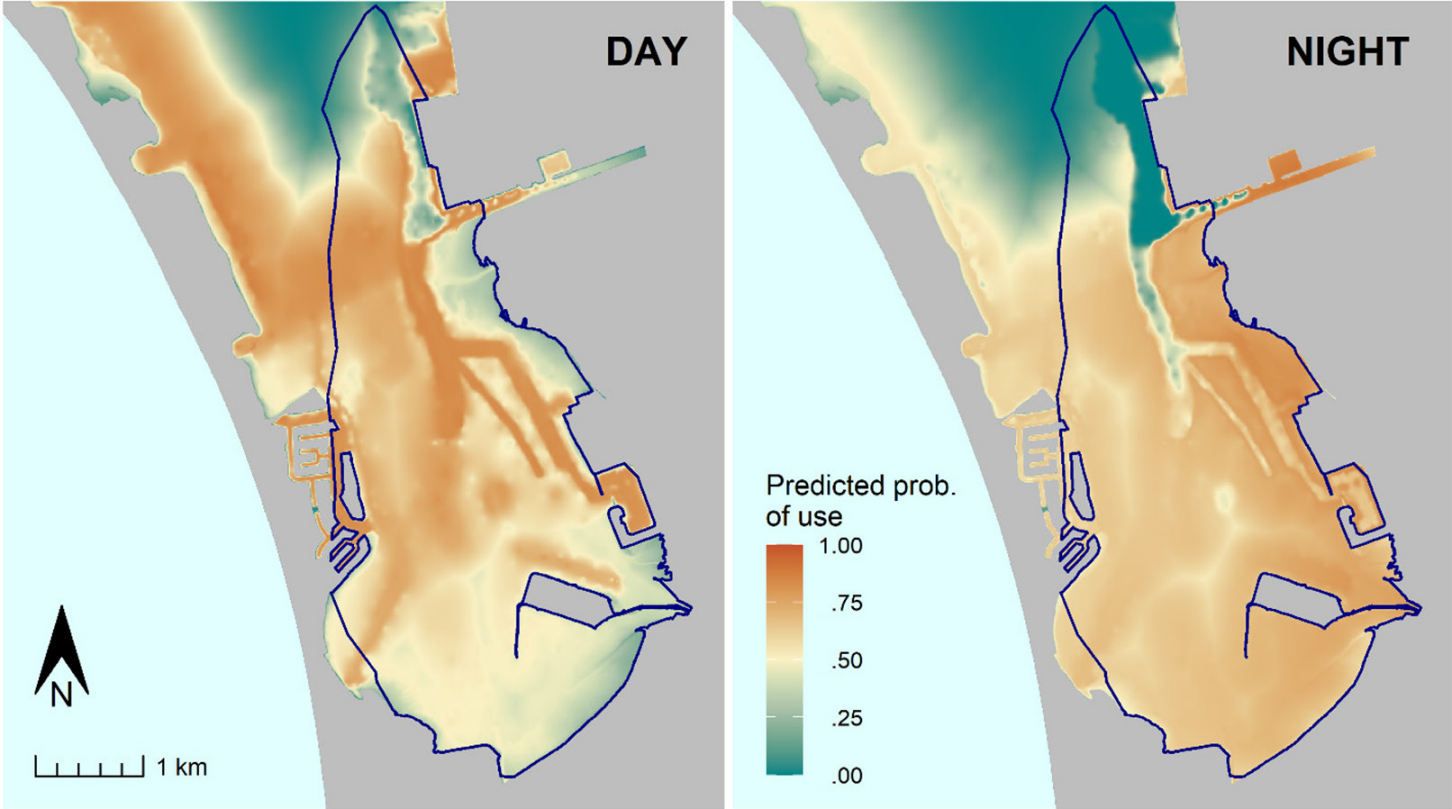
Spatial predictions of probability of use by green turtles (*Chelonia mydas*) based on the best‐performing model of warm‐season resource selection in south San Diego Bay. The dark blue polygon outline shows the true area of inference (i.e., the “available area”)—the joint‐99% utilization distribution. Predictions were made using input raster data consisting of bathymetric water depth, water temperatures, and the 2020 eelgrass coverage (Figure 3) for a turtle of mean body size (80.9 cm SCL). 95% prediction intervals for raster cell predictions, which ranged <0.001–0.24 for the day and <0.001–0.32 for the night, are mapped in space in Appendix 6 (Figure A4).

**FIGURE A4:**
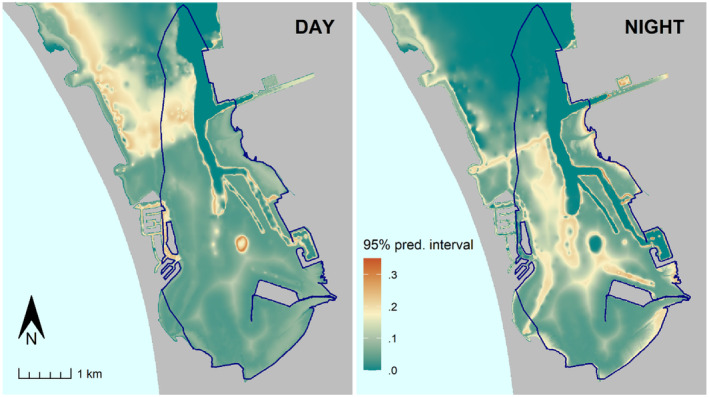
Uncertainty associated with spatial predictions of warm‐season probability of use by green turtles (*Chelonia mydas*) south San Diego Bay. See Figure 7 for predicted probabilities. Values, depicted with a blue to red gradient, show the width of the 95% prediction interval, with higher values associated with greater uncertainty. The dark blue polygon outline shows the true area of inference (i.e., the “available area”)—the joint‐99% utilization distribution. Predictions were made using input raster data consisting of bathymetric water depth, water temperatures, and the 2020 eelgrass coverage (Figure 4) for a turtle of mean body size (80.9 cm SCL) in the warm season. Intervals spanned <0.001–0.24 for the day and <0.001–0.32 for the night.

Model structures, AICc scores, and parameter estimates are detailed in Appendix 4. Given complicated models with two‐ and three‐way interactions, variable effects and interactions are best interpreted visually. We illustrate effects for selected warm‐ and cold‐season models in Figures 5 and 6, respectively, and elaborate on patterns in the discussion. Warm‐season model findings are also displayed spatially in Figure 7, which contrasts daytime versus nighttime predicted probability of use for a turtle of mean body size. Uncertainty associated with these spatial predictions is mapped in Appendix 6 (Figure A4).

The warm‐season model comparison considered 25 models in total. After phase one of comparisons (no interaction terms), we determined that quadratic effects for depth and distance to eelgrass edge aided model performance and we accordingly conditioned all subsequent models to contain these terms. Phase two revealed that day–night interactions with spatial covariates improved models more than interactions with body size, and we included interactions between day–night and all spatial covariates when designating models for phase three. The third and final phase of model comparison provided support for three‐way interactions among body size, day–night, and two resource variables: depth and relative water temperature (Table A2). The top‐performing warm‐season model had a pseudo‐*R* ^2^ value of .087 and received 88% of the AICc weight, with the second‐ranking model receiving ~12% and all other models <0.01% (Table A1). The Type II analysis of deviance for the top model suggested that all but two terms significantly improved model fit (*p* < .05; Table A3).

Phased comparisons of 25 cold‐season models resulted in distinct candidate model structures for consideration (Table A4). As with warm‐season models, phase one suggested that all spatial covariates improved fit. Phase two then diverged from the warm‐season comparison exercise, revealing that interactions between body size (SCL) and spatial covariates improved models more than interactions with day–night, with interactions supported with all three spatial predictors. Finally, phase three revealed a lack of support for any three‐way interactions, and the top‐performing model ended up being the best model from phase two (Table A5). The selected cold‐season model had a pseudo‐*R* ^2^ of .203, receiving 55% of the AICc weight. The second‐ranking model received 22% and all other models received <10% (Table A4). In this case the selected model only contained two‐way interactions between continuous terms, and as such we did not perform a Type II analysis of deviance (i.e., relevant information can be readily obtained from the model fit; Table A5).

## 4 DISCUSSION

Our study leveraged a long‐term satellite telemetry dataset and high‐resolution habitat data to provide new insight into resource selection by foraging green turtles. The selection patterns that we documented suggest three broad takeaways. First, during the warmest 8 months of the year (Apr–Nov), green turtles at our study site exhibited daytime selection for areas well within seagrass patches; however, the peak in selection moved closer to, and outside of, the patch edge at night (Figure 5). This behavior was shown consistently across years and individuals of different sizes. Second, during warm months, turtles displayed clear patterns in fine‐scale depth and temperature selection that were modulated by both turtle body size and diel period (day vs. night; Figure 5). Third, limited cold‐season data indicated that patterns in resource selection may vary seasonally—observed behavior did not vary by day and night during the coldest months, when turtles consistently selected for shallow, warm areas, with more nuanced variation in seagrass use according to body size (Figure 6). These patterns can help managers implement measures to support green turtle foraging populations and provide insight into the influence of environmental resources at a uniquely fine scale (i.e., within a foraging home range).

Our work additionally presents a broadly applicable methodological approach for modeling spatial resource selection with telemetry data for aquatic species. By accounting for the Fastloc‐GPS error and autocorrelation associated with each location fix, we were able to minimize bias while informing models with point locations. This preserved interesting, fine‐scale patterns in space use that other approaches miss, notably those that analyze selection only after integrating GPS points into a home range. Fine‐scale inferences were made possible by the availability of high‐resolution habitat data, for example triennial eelgrass coverages (Merkel & Associates, 2020), which underscores the value of allocating resources toward describing habitats at long‐term research sites. Future work could improve on our analytical methods by incorporating Fastloc‐GPS errors measured at the study area, rather than from a previous study elsewhere (Dujon et al., 2014), and/or by employing more sophisticated modeling frameworks such as integrated RSFs (Alston et al., 2023), especially when such approaches are extended to account for telemetry error.

### 4.1 Environmental drivers of fine‐scale space use

#### 4.1.1 Patterns in seagrass selection

Our results demonstrate selection for seagrass habitat during the warmest months in SDB, adding to the body of evidence establishing spatial associations between omnivorous green turtles and seagrass meadows (e.g., Griffin et al., 2020). Seagrasses comprise a global species group that has suffered human‐driven declines (Orth et al., 2006), but for which management and recovery efforts hold significant promise (Buelow et al., 2022). We make an important advance by quantifying seagrass association for green turtles in the Eastern Pacific, a region in which populations tend to exhibit weak dietary associations with the resource (e.g., Lemons et al., 2011) and in which relatively few studies have elucidated drivers of foraging movements (Senko et al., 2010). Our study suggests that when seagrass is available, East Pacific green turtles will readily utilize it. Green turtles in SDB particularly inhabit meadows of the eelgrass *Zostera marina*, an ecosystem engineer supporting diverse energy pathways for consumers (Orth et al., 2006)—the eelgrass itself, plus epiphytic algae, habitat‐forming macroalgae, and epifaunal consumers (Lemons et al., 2011; Sirota & Hovel, 2006). Eelgrass is highly dynamic in the northeastern Pacific (Munsch et al., 2023) and garners significant management attention. Our findings help to justify this attention and, moreover, support its designation as critical habitat under the United States Endangered Species Act (National Marine Fisheries Service, 2023).

Beyond a general finding of eelgrass association, finer‐scale patterns revealed variation in eelgrass selection by day and night in addition to season. We did not find support for variation among body sizes in the warm season, as all turtles appeared to utilize eelgrass beds similarly (in terms of distance from eelgrass edges). In the warm season, turtles exhibited much stronger selection for eelgrass during the day. After controlling for other variables, daytime predicted probability of selection was at its maximum well into the interior of eelgrass habitats (~250 m inside edges; Figure 5). At night, the maximum shifted to ~90 m outside of edges. By contrast, in the cold season, a period for which we had far fewer data, there was no evidence for day–night differences. Turtles generally selected for areas outside of eelgrass habitats, with the exception of the largest individual, which appeared to use areas either well inside or outside the eelgrass edge. We caution against drawing significant conclusions for size‐based patterns in the cold season, as with only five individuals tracked, patterns may be an artifact individual behavior. We also reiterate that given patterns in patch dynamics (e.g., expansion, retreat) documented for regional eelgrass on annual timescales (Munsch et al., 2023), there was likely nontrivial variation in eelgrass edge location in between triennial eelgrass surveys that we were not able to model. As such, the location of selection maxima, etc., are best used to interpret model output, not to provide precise management guidance.

Day–night shifts in warm‐season eelgrass association are most likely related to diel patterns in foraging. That is, assuming prey species are concentrated within eelgrass meadows, turtles may eat more during the day as compared to night, a pattern suggested by previous research in SDB (MacDonald et al., 2013) and elsewhere (Christiansen et al., 2016; Ogden et al., 1983). There was less evidence for eelgrass selection in the cold season, including a lack of any day–night differences. Although based on a limited sample size, this finding could be explained by reduced metabolic activity in colder water (Litzgus & Hopkins, 2003) leading to less foraging overall and thus less frequent visitation of eelgrass meadows. Alternatively, prey selection and/or distribution may change with season. For example, eelgrass meadows can exhibit seasonal changes in biomass (Hasegawa et al., 2007), and although our anecdotal observations in SDB suggest that foraging resources are concentrated within eelgrass beds throughout all seasons, green turtles may have sufficient dietary plasticity such that they could switch primary prey seasonally in dynamic, temperate systems. Green turtles in Southern California can exhibit year‐round residency in a foraging habitat and are subjected to some of the coldest winter water temperatures documented for the species (as low as 15.8°C; Hanna et al., 2021); however, seasonal differences in foraging activity in temperate areas have not received significant attention. Previous work has demonstrated clear behavioral changes by green turtles in cold water, notably a shift to dives of longer duration (Hazel et al., 2009; Madrak et al., 2022), that are consistent with reduced metabolic rates. The behavior we observed of reduced eelgrass selection during cold months fits this pattern, though additional cold‐season tracking is needed before drawing strong conclusions.

#### 4.1.2 Depth and temperature selection

Water depth and surface temperatures are inherently linked, as deeper areas generally feature cooler temperatures (a relationship indeed indicated by our spatial data in SDB). We note that these variables were not excessively correlated per our a priori criteria for modeling and, further, highlight that key areas of discordance exist in South Bay (Figure 3e,f). Nonetheless, we discuss depth and temperature coincidently given this clear relationship. A basic pattern that emerged from warm‐season data entailed turtles shifting into shallower, warmer areas at night (Figure 7), and the degree of this shift was modulated by body size (Figure 5). The largest turtles selected for the deepest and coolest habitats during the day when, conversely, the smallest turtles selected for much warmer and shallower areas. All turtles selected for similarly shallow, warm areas at night, entailing a larger habitat shift for bigger turtles. For example, after controlling for other variables, warm‐season model predictions suggested that a turtle two standard deviations larger than the mean would shift maximum probability of selection from a bathymetric depth of 7 m during the day to 2.4 m at night. By contrast, a turtle two SDs smaller than the mean shifted by just 1.4 m in depth, from 4 m to 2.6 m. In the cold season, however, diel shifts were again absent per our modeling results (*N* = 5 individuals tracked during this season). Size‐based differences did materialize in the cold season, with a general pattern of smaller turtles consistently exhibiting stronger selection for shallower, warmer habitats.

The diel movement patterns we observed could be related to factors such as thermoregulation, visitation of foraging versus resting habitats, or human avoidance. Given all evidence, thermoregulation may be the most plausible explanation. Larger turtles should have increased thermoregulatory capacity (Sato et al., 1998), allowing them to access deeper, cooler areas during the day within relatively warm months and/or driving them to seek these areas to cool down. Large individuals then move to shallow, warm habitats at night when daily temperatures drop. In contrast to warm‐season findings, limited inferences into cold‐season behavior indicate that all turtles selected for shallow, warm areas during both night and day. As mentioned previously, SDB is located near the northern limit of the Eastern Pacific range of green turtles, presenting winter water temperatures that are extremely cold relative to global habitats generally occupied by the species (Esteban et al., 2020). Thus, as metabolic rates slow, turtles of all sizes may prioritize thermoregulation in warm, shallow habitats.

Similar seasonal selection for warm water is consistent with previous research on loggerheads (*Caretta caretta*) in the Mediterranean (Schofield et al., 2009) and Northwest Atlantic (Hawkes et al., 2011), albeit at different spatial scales. It is also possible that diel shifts in depth selection were a function of the distribution of rest areas, as nighttime resting in shallow habitats has been documented for green turtles at our study site (MacDonald et al., 2013) and other locations (Chambault et al., 2020; Christiansen et al., 2016; Crear et al., 2017). However, there are other conflicting lines of evidence for the SDB aggregation suggesting that activity may not decline at night—our GPS data reflect similar rates of surfacing by night and day, and Eguchi et al. (2020) documented similar home range sizes in both night and day. Further investigation would be beneficial to determine whether shallow areas are indeed used for rest at night. Finally, human avoidance may also play a role in diel shifts within urban SDB. That is, turtles may move to deeper habitats during the day to avoid daytime peaks in human activity, such as boat traffic. However, given the absence of a deep‐shallow shift in the cold season, thermoregulation seems like a more likely driver than human avoidance. We note that previous studies have described contrasting shifts whereby green turtles moved to deeper habitats at night (Griffin et al., 2020; Hazel, 2009). Therefore, day–night shifts may be specific to a given area's thermal regime, habitat composition, and potentially predation environment—a factor largely absent in SDB.

### 4.2 Scale of inference

The primary extent of inference for this study was relatively small, encapsulating 12.4 km^2^ of habitat at the southern extreme of SDB. How our findings apply to other contexts merits further study, though we offer some potentially generalizable results, such as the takeaway that foraging movements clearly vary with body size in a temperate system. In Southern California, green turtle populations appear to be increasing per multiple lines of evidence, including sightings, strandings, and mark–recapture sampling (NOAA unpubl. data). This trend is consistent with increased reproductive output on nesting beaches in Pacific Mexico in conjunction with elevated fisheries protections regionally (Delgado‐Trejo & Alvarado‐Diaz, 2012). Thus, management challenges associated with mitigating human impacts at foraging areas are also increasing proportionally. We suggest that SDB can serve as a model system for studying green turtle ecology and behavior to inform management in this changing region. We also highlight that there is much left to learn about resource selection in our system (and others). There were many factors that we were not able to model—including intra‐ and inter‐specific interactions, human disturbance, individual behavioral variation, and others—as reflected by pseudo‐*R* ^2^ values suggesting we could explain just 9% of the variance in selection in the warm season, when 85% of our data were collected, and 20% during the cold season.

Our study reveals drivers of fine‐scale movements within a home range, i.e., after the conditions for the selection the home range location were met. Thus, key questions remain regarding the drivers of broader “habitat selection,” that is, the placement of foraging home ranges on the landscape. This process is likely relatively deterministic, largely constrained by physiology and resource availability. Inference into habitat selection would be best derived via distribution modeling integrating environmental data over a much larger spatial extent (e.g., Lopez et al., 2024). We suggest that it would be particularly interesting to discern the role of eelgrass in the spatially broader process of habitat selection. While eelgrass is a driver of fine‐scale resource selection per our findings in a relatively eelgrass‐dominated system, green turtles could potentially utilize other foraging resources such as algae, as exemplified by the presence of a foraging aggregation in a nearby area largely devoid of seagrass (San Gabriel River, California, ~160 km NNW of SDB; Massey et al., 2023). Such evidence poses the conjectural possibility that green turtles in the region simply require relatively warm, productive water. Yet, turtles also inhabit areas that stay relatively cold for much of the year (i.e., La Jolla Shores, California; Hanna et al., 2021), albeit at low densities, additionally demonstrating that individuals are able to adapt to different thermal environments and thus may simply require resource productivity (not necessarily warm water). However, these examples fall short of insight into preferences relative to availability, and we conclude future work employing distribution models would clearly be beneficial for the west coast of North America in a context of population expansion.

## 5 CONCLUSIONS

Our study provides quantitative habitat associations representing actionable information for managers tasked with conserving sea turtles in their foraging habitats. Resulting insights into resource selection have potential applicability for any sea turtle species. For example, we document interesting diel shifts in selection that varied with both body size and season. Understanding such shifts can aid spatial conservation planning and enhance our understanding of foraging ecology. More specifically, the findings we present can inform green turtle conservation within the complex management context of Southern California. This region hosts dense coastal urbanization and therefore high levels of anthropogenic impacts—ranging from coastal construction, to noise pollution, fisheries interactions, and boat traffic. Coastal development is only expanding. For instance, at the time of this study, work on a recently approved, billion‐dollar waterfront development was underway in South Bay (in Chula Vista, California) immediately adjacent to the study site, which we expect will lead to effects on turtles such as increased anthropogenic noise and boat‐based recreation. More broadly, the unique inferences we offer come at a pivotal time within the eastern North Pacific, where available evidence suggests that green turtle numbers are increasing. Within this regional context of population expansion, under increasingly urban circumstances in some cases, detailed knowledge of diel and seasonal movement tendencies may be integrated into policy measures designed to reduce human impacts, such as speed limits or protected area designation.

## AUTHOR CONTRIBUTIONS

**Andrew S. Maurer:** Conceptualization (lead); formal analysis (lead); investigation (supporting); writing – original draft (lead); writing – review and editing (lead). **Tomo Eguchi:** Data curation (supporting); investigation (supporting); writing – review and editing (supporting). **Garrett E. Lemons:** Data curation (supporting); investigation (supporting); project administration (equal); writing – review and editing (supporting). **Robin A. LeRoux:** Investigation (supporting); project administration (equal); writing – review and editing (supporting). **Erin L. LaCasella:** Investigation (supporting); writing – review and editing (supporting). **Calandra N. Turner Tomaszewicz:** Investigation (supporting); writing – review and editing (supporting). **Megan E. Hanna:** Investigation (supporting); resources (supporting); writing – review and editing (supporting). **Jessica Curran:** Investigation (supporting); resources (supporting); writing – review and editing (supporting). **Bryant Chesney:** Investigation (supporting); writing – review and editing (supporting). **Sheila V. Madrak:** Investigation (supporting); writing – review and editing (supporting). **Jeffrey A. Seminoff:** Conceptualization (supporting); data curation (lead); investigation (lead); project administration (equal); writing – original draft (supporting); writing – review and editing (supporting).

## CONFLICT OF INTEREST STATEMENT

The authors declare no conflicts of interest.

## Supporting information


Data S1

Data S2

Data S3

## Figures and Tables

**FIGURE A1:**
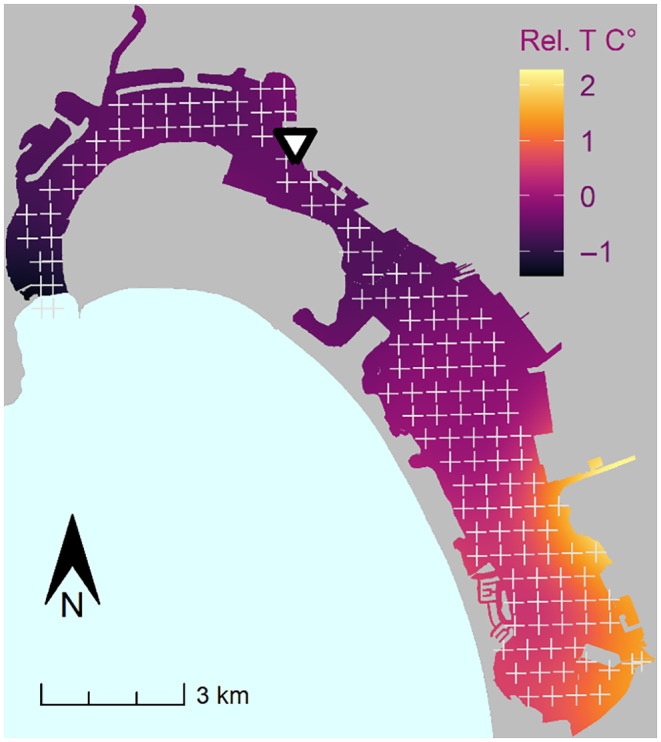
Results of ordinary kriging interpolation of mean relative water temperatures in San Diego Bay. Crosses display the locations of 133 sampling stations used by Madrak et al. (2016). The white triangle marks the location of NOAA data buoy SDBC1. Kriging results have been masked to the inner shoreline of the bay.

**FIGURE A2:**
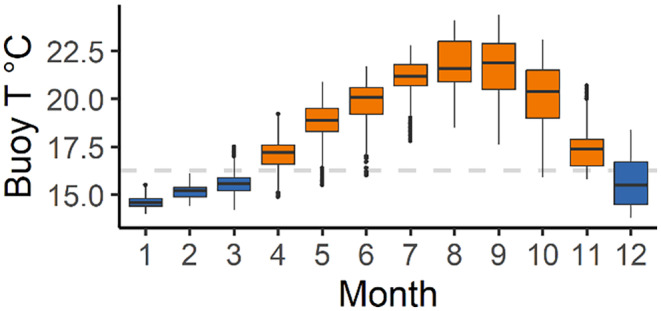
Monthly water temperatures at NOAA buoy SDBC1 in San Diego Bay over 2011–2012. Boxes depict the monthly median and first and third quartiles, while whiskers extend to the largest and smallest values no further than 1.5 times the inter‐quartile range from the associated quartile. The dashed gray line delineates the seasonal delineation threshold—the coldest four months, shown in blue, were designated as the cold season, as compared to the warm season in orange.

## Data Availability

The data supporting the findings of this study are available in the supplementary material of this article.

## APPENDIX 1 Interpolating the spatial distribution of mean water temperatures in San Diego Bay

### 1.1

Interpolation entails using known values at a given set of points to estimate unknown values for the space around those points. One can generate estimates for a variable in continuous 2D space at a chosen resolution (i.e., a raster surface) based on georeferenced point measurements of that variable. We interpolated a temperature surface for San Diego Bay based on water temperatures repeatedly measured at a depth of 1 m at 113 stations located in the center of 500‐m grid cells overlaid on the bay (Figure A1). Madrak et al. (2016) surveyed these grid locations repeatedly in 2011 (after powerplant closure), collecting a mean ± SD of 5.70 ± 1.9 measurements per station. South Bay was emphasized in sampling, with the southernmost 48 stations visited 7.17 ± 2.5 times. Importantly, these measurements were collected at irregular intervals, and only a subset of locations was surveyed on each sampling occasion. Thus, we could not calculate simple mean temperatures, as measurements were temporally irregular and water temperatures can vary substantially on daily scales. As such, we related grid stations to the local NOAA data buoy SDBC1, which conveniently records a measurement at regular 6‐min intervals. We fit a linear regression with grid station temperature (for a given sampling occasion) as the response term and two predictor variables: (1) a continuous term for mean buoy temperature for the day of sampling and (2) a factorial term for grid station (1–133). The intercept estimates for each station provided a mean difference in temperature from the buoy. We recentered the 133 intercept estimates to a mean of 0 and used the values (referred to as “relative water temperatures” herein) for interpolation, with the values ranging −2.10 to 3.60°C (SD = 0.76°C). We assumed these relative temperatures (i.e., the distribution of relatively warm and cold areas in San Diego Bay) would remain relatively consistent through time, and thus applied the 2011 data for the entire study period.

We used ordinary kriging to interpolate a raster surface from the georeferenced relative water temperature values with the R package gstat (Gräler et al., 2016). We selected kriging over two other methods because its output better matched our expectations given multiple decades of research in the system. Other methods considered included inverse‐distance weighted interpolation with a null model, also using gstat (Gräler et al., 2016), and thin plate spline interpolation with default parameter settings in the fields R package (Nychka et al., 2021). To execute ordinary kriging, we first calculated direct and cross‐sample variograms from the data, setting the cutoff and width parameters to 7200 m and 480 m, respectively (the bounding box for the 133 stations was 1296 m wide east‐west and 12,814 m north‐south). We then fit a Gaussian model variogram with initial parameter values for the partial sill set to 0.45, the range to 5000, and the nugget to 0.1. The sill estimates for the resulting model variogram were 0.0668 for the nugget and 0.370 for the Gaussian portion of the model, while the range estimates were 0 and 2557.2, respectively. We used this variogram fit to conduct ordinary kriging over the extent and resolution of our bathymetry layer (Figure A1). The estimated surface was then used to annotate temperature values onto point locations for resource selection functions.

## APPENDIX 2 Delineating seasons relevant to foraging ecology

### 2.1

Sea turtles are ectothermic, and thus, environmental temperatures have a major role in their physiology and behavior (Madrak et al., 2022; Schofield et al., 2009; Zuo et al., 2011). Accordingly, we accounted for seasonal differences in water temperatures in our analysis of resource selection. To delineate two separate seasons (i.e., relatively warm versus cold), we calculated monthly mean water temperatures using measurements recorded at 6‐min intervals at the local NOAA buoy SDBC1 over 2011–2012 (Figure A2), a period after the closure of the warm effluent‐producing powerplant and before the appearance of erroneously high‐temperature anomalies in the buoy data that began in 2013. We note that these buoy temperatures are considerably colder than the primary green turtle habitat in southern SDB, but nonetheless offer a time series for seasonal differentiation (Figure A1). We chose a threshold identifying the four coldest months based on years of experience working in the study system, and although this choice is in effect arbitrary, it provides a break to explore seasonal effects. It is likely that a discrete effect from warm versus cold seasons is only a coarse proxy for what is truly a continuous effect from water temperature. This is beyond the scope of our analysis because, given our modeling framework, exploring an effect at such a temporal resolution would compromise spatial information in our data. Nonetheless, more research exploring the effect of continuous water temperatures on habitat use would be a worthy pursuit.

## APPENDIX 3 Accounting for autocorrelation: movement modeling and autocorrelation weighting

### 3.1

We down‐weighted autocorrelated points following workflows suggested for package ctmm in R, and specifically function akde (Calabrese et al., 2016). For each individual's set of GPS locations, we first calculated an empirical variogram, and then used that variogram to inform selection of a best‐fitting continuous time movement model (on the basis of AICc). This type of movement model was appropriate because we only considered individuals observed exhibiting nonmigratory behavior. Models were fit with perturbative hybrid residual maximum likelihood, and selected models fell within one of six prototype classes: independent and identically distributed bivariate Gaussian (IID), IID anisotropic, Brownian motion restricted to a finite home range (Ornstein–Uhlenbeck), Ornstein–Uhlenbeck anisotropic, continuous‐velocity motion restricted to a finite home range (OUF), OUF anisotropic, or OUf anisotropic (similar to OUF anisotropic but with slight deviations). For more details on these models and the model fitting workflow, see the package documentation for ctmm (https://cran.r‐project.org/package=ctmm) and the references provided there. We next used the continuous time movement model fit for each individual to estimate its effective sample size and compute autocorrelation weights using the akde function. We used autocorrelation weights and effective sample sizes to inform a model weight term as described in the Methods section (section 2.2.3).

## APPENDIX 4 Modeling results

**TABLE A1:** Comparison of 25 logistic models of resource selection by green turtles (*Chelonia mydas*; *N* = 28 individuals) during the warm season, including assessment of size‐specific and day–night differences in behavior (*N* = 4274 GPS locations and 4257 pseudo‐absences).

Warm‐season model structure	Phase	*K*	ΔAICc	ω
d.eelgrass + d.eelgrass^2^ + day‐night * (d.eelgrass + d.eelgrass^2^) + depth + depth^2^ + day‐night * SCL * (depth + depth^2^) + T° + day‐night * SCL * T°	3	14	0	0.88
d.eelgrass + d.eelgrass^2^ + day‐night * SCL * (d.eelgrass + d.eelgrass^2^) + depth + depth^2^ + day‐night * SCL * (depth + depth^2^) + T° + day‐night * SCL * T°	3	16	3.99	0.12
d.eelgrass + d.eelgrass^2^ + day‐night * (d.eelgrass + d.eelgrass^2^) + depth + depth^2^ + day‐night * SCL * (depth + depth^2^) + T° + day‐night * T°	3	13	20.6	0
d.eelgrass + d.eelgrass^2^ + day‐night * SCL * (d.eelgrass + d.eelgrass^2^) + depth + depth^2^ + day‐night * SCL * (depth + depth^2^) + T° + day‐night * T°	3	15	25.8	0
d.eelgrass + d.eelgrass^2^ + day‐night * (d.eelgrass + d.eelgrass^2^) + depth + depth^2^ + day‐night * (depth + depth^2^) + T° + day‐night * SCL * T°	3	12	37.2	0
d.eelgrass + d.eelgrass^2^ + day‐night * (d.eelgrass + d.eelgrass^2^) + depth + depth^2^ + day‐night * (depth + depth^2^) + T° + day‐night * T°	2	11	37.8	0
d.eelgrass + d.eelgrass^2^ + day‐night * SCL * (d.eelgrass + d.eelgrass^2^) + depth + depth^2^ + day‐night * (depth + depth^2^) + T° + day‐night * SCL * T°	3	14	43.7	0
d.eelgrass + d.eelgrass^2^ + day‐night * SCL * (d.eelgrass + d.eelgrass^2^) + depth + depth^2^ + day‐night * (depth + depth^2^) + T° + day‐night * T°	3	13	44.4	0
d.eelgrass + d.eelgrass^2^ + depth + depth^2^ + day‐night * (depth + depth^2^) + T° + day‐night * T°	2	9	72.9	0
d.eelgrass + d.eelgrass^2^ + day‐night * (d.eelgrass + d.eelgrass^2^) + depth + depth^2^ + day‐night * (depth + depth^2^) + T°	2	10	193	0
d.eelgrass + d.eelgrass^2^ + depth + depth^2^ + day‐night * (depth + depth^2^) + T°	2	8	225	0
d.eelgrass + d.eelgrass^2^ + day‐night * (d.eelgrass + d.eelgrass^2^) + depth + depth^2^ + T° + day‐night * T°	2	9	259	0
d.eelgrass + d.eelgrass^2^ + depth + depth^2^ + T° + day‐night * T°	2	7	301	0
d.eelgrass + d.eelgrass^2^ + day‐night * (d.eelgrass + d.eelgrass^2^) + depth + depth^2^ + T°	2	8	305	0
d.eelgrass + d.eelgrass^2^ + depth + depth^2^ + SCL * (depth + depth^2^) + T° + SCL * T°	2	9	309	0
d.eelgrass + d.eelgrass^2^ + SCL * (d.eelgrass + d.eelgrass^2^) + depth + depth^2^ + SCL * (depth + depth^2^) + T° + SCL * T°	2	11	314	0
d.eelgrass + d.eelgrass^2^ + depth + depth^2^ + SCL * (depth + depth^2^) + T°	2	8	315	0
d.eelgrass + d.eelgrass^2^ + SCL * (d.eelgrass + d.eelgrass^2^) + depth + depth^2^ + SCL * (depth + depth^2^) + T	2	10	319	0
d.eelgrass + d.eelgrass^2^ + depth + depth^2^ + T° + SCL * T°	2	7	329	0
d.eelgrass + d.eelgrass^2^ + depth + depth^2^ + T°	1	6	331	0
d.eelgrass + d.eelgrass^2^ + SCL * (d.eelgrass + d.eelgrass^2^) + depth + depth^2^ + T° + SCL * T°	2	9	334	0
d.eelgrass + d.eelgrass^2^ + SCL * (d.eelgrass + d.eelgrass^2^) + depth + depth^2^ + T°	2	8	336	0
d.eelgrass + depth + depth^2^ + T°	1	5	440	0
d.eelgrass + d.eelgrass^2^ + depth + T°	1	5	604	0
d.eelgrass + depth + T°	1	4	768	0

*Note*: Pseudo‐absences were generated within an available area designated by the joint‐99% utilization distribution for all turtles tracked in the study (*N* = 29). Phase refers to one of three phases of model comparison (see Methods Section 2.2.4). *K* is the number of parameters estimated by the model, ΔAICc is the difference in AICc score, and the AICc weight ω reflects the probability of a model being the most parsimonious out of all considered. Terms are defined as follows: d.eelgrass denotes distance to the nearest eelgrass patch edge, depth denotes bathymetric water depth, T° denotes mean relative sea surface temperature, day–night denotes a binary day–night term, and SCL denotes straight carapace length (i.e., body size).

**TABLE A2:** Parameter estimates for the top‐performing warm‐season model of resource selection by green turtles (*Chelonia mydas*; *N* = 29 individuals) at the scale of the joint‐99% home range.

Parameter	Coefficient est.	SE	*p*
Intercept	−1.75 × 10^−1^	1.11 × 10^−1^	.116
d.eelgrass	−9.72 × 10^−4^	2.68 × 10^−4^	<.001
d.eelgrass^2^	−1.94 × 10^−6^	4.30 × 10^−7^	<.001
depth	−4.47 × 10^−1^	1.35 × 10^−1^	<.001
depth^2^	−8.40 × 10^−2^	1.97 × 10^−2^	<.001
T°	9.22 × 10^−1^	1.81 × 10^−1^	<.001
d.eelgrass * night	1.91 × 10^−3^	3.80 × 10^−4^	<.001
d.eelgrass^2^ * night	−3.32 × 10^−6^	7.52 × 10^−7^	<.001
depth * day * SCL	−3.01 × 10^−3^	1.61 × 10^−3^	.061
depth * night * SCL	3.43 × 10^−4^	1.69 × 10^−3^	.839
depth^2^ * day * SCL	2.36 × 10^−4^	2.28 × 10^−4^	.301
depth^2^ * night * SCL	1.54 × 10^−5^	2.66 × 10^−4^	.954
T° * day * SCL	−1.08 × 10^−2^	2.06 × 10^−3^	<.001
T° * night * SCL	−9.11 × 10^−4^	2.00 × 10^−3^	.649

*Note*: The variance function‐based pseudo‐*R* ^2^ was .087.

**TABLE A3:** Results from a Type II analysis of deviance for the best‐performing warm‐season model of resource selection. Model selection statistics are displayed in Table A1 and coefficient estimates are reported in Table A2.

Model term	*df*	*p*
d.eelgrass	1	.629
d.eelgrass^2^	1	<.001
depth	1	<.001
depth^2^	1	<.001
T°	1	<.001
d.eelgrass * day‐night	1	<.001
d.eelgrass^2^ * daynight	1	<.001
depth * day‐night * SCL	2	<.001
depth^2^ * day‐night * SCL	2	.174
T° * day‐night * SCL	2	<.001

**TABLE A4:** Comparison of 25 logistic models of resource selection during the cold season (*N* = 749 GPS locations and 750 pseudo‐absences) for five green turtles (*Chelonia mydas*).

Cold‐season model structure	Phase	*K*	ΔAICc	ω
d.eelgrass + d.eelgrass^2^ + SCL * (d.eelgrass + d.eelgrass^2^) + depth + depth^2^ + SCL * (depth + depth^2^) + T° + SCL * T°	2	11	0	0.55
d.eelgrass + d.eelgrass^2^ + SCL * (d.eelgrass + d.eelgrass^2^) + depth + depth^2^ + SCL * day‐night * (depth + depth^2^) + T° + day‐night * SCL * T°	3	12	1.87	0.22
d.eelgrass + d.eelgrass^2^ + SCL * (d.eelgrass + d.eelgrass^2^) + depth + depth^2^ + day‐night * SCL * (depth + depth^2^) + T° + SCL * T°	3	13	3.5	0.09
d.eelgrass + d.eelgrass^2^ + day‐night * SCL * (d.eelgrass + d.eelgrass^2^) + depth + depth^2^ + SCL * (depth + depth^2^) + T° + SCL * T°	3	13	4.68	0.05
d.eelgrass + d.eelgrass^2^ + SCL * (d.eelgrass + d.eelgrass^2^) + depth + depth^2^ + day‐night * SCL * (depth + depth^2^) + T° + day‐night * SCL * T°	3	14	5.53	0.03
d.eelgrass + d.eelgrass^2^ + day‐night * SCL * (d.eelgrass + d.eelgrass^2^) + depth + depth^2^ + SCL * (depth + depth^2^) + T° + day‐night * SCL * T°	3	14	6.45	0.02
d.eelgrass + d.eelgrass^2^ + SCL * (d.eelgrass + d.eelgrass^2^) + depth + depth^2^ + SCL * (depth + depth^2^) + T°	2	10	6.66	0.02
d.eelgrass + d.eelgrass^2^ + day‐night * SCL * (d.eelgrass + d.eelgrass^2^) + depth + depth^2^ + day‐night * SCL * (depth + depth^2^) + T° + SCL * T°	3	15	8.09	0.01
d.eelgrass + d.eelgrass^2^ + day‐night * SCL * (d.eelgrass + d.eelgrass^2^) + depth + depth^2^ + day‐night * SCL * (depth + depth^2^) + T° + day‐night * SCL * T°	3	16	10.1	0
d.eelgrass + d.eelgrass^2^ + SCL * (d.eelgrass + d.eelgrass^2^) + depth + depth^2^ + T°	2	8	12.9	0
d.eelgrass + d.eelgrass^2^ + SCL * (d.eelgrass + d.eelgrass^2^) + depth + depth^2^ + T° + SCL * T°	2	9	14.7	0
d.eelgrass + d.eelgrass^2^ + depth + depth^2^ + SCL * (depth + depth^2^) + T° + SCL * T°	2	9	35.8	0
d.eelgrass + d.eelgrass^2^ + depth + depth^2^ + T°	1	6	41.9	0
d.eelgrass + d.eelgrass^2^ + depth + depth^2^ + T° + SCL * T°	2	7	42.1	0
d.eelgrass + d.eelgrass^2^ + depth + depth^2^ + SCL * (depth + depth^2^) + T°	2	8	42.3	0
d.eelgrass + d.eelgrass^2^ + depth + depth^2^ + T° + day‐night * T°	2	7	43.7	0
d.eelgrass + d.eelgrass^2^ + depth + depth^2^ + day‐night * (depth + depth^2^) + T°	2	8	45.1	0
d.eelgrass + d.eelgrass^2^ + day‐night * (d.eelgrass + d.eelgrass^2^) + depth + depth^2^ + T°	2	8	45.4	0
d.eelgrass + d.eelgrass^2^ + depth + depth^2^ + day‐night * (depth + depth^2^) + T° + day‐night * T°	2	9	47.0	0
d.eelgrass + d.eelgrass^2^ + day‐night * (d.eelgrass + d.eelgrass^2^) + depth + depth^2^ + T° + day‐night * T°	2	9	47.1	0
d.eelgrass + d.eelgrass^2^ + depth + T°	1	5	47.5	0
d.eelgrass + d.eelgrass^2^ + day‐night * (d.eelgrass + d.eelgrass^2^) + depth + depth^2^ + day‐night * (depth + depth^2^) + T°	2	10	49.0	0
d.eelgrass + d.eelgrass^2^ + day‐night * (d.eelgrass + d.eelgrass^2^) + depth + depth^2^ + day‐night * (depth + depth^2^) + T° + day‐night * T°	2	11	50.8	0
d.eelgrass + depth + depth^2^ + T°	1	5	56.3	0
d.eelgrass + depth + T°	1	4	64.2	0

*Note*: Pseudo‐absences were generated within the joint‐99% utilization distribution. Terms and naming conventions match those of Table A3.

**TABLE A5:** Parameter estimates for the top‐performing cold‐season model of resource selection by green turtles (*Chelonia mydas*; *N* = 5 individuals) at the scale of the joint‐99% home range (pseudo‐*R* ^2^ = .203).

Parameter	Coefficient est.	SE	*p*
Intercept	−1.09	2.77 × 10^−1^	<.001
d.eelgrass	1.94 × 10^−2^	3.62 × 10^−3^	<.001
d.eelgrass^2^	−4.00 × 10^−5^	7.82 × 10^−6^	<.001
depth	−1.96	5.34 × 10^−1^	<.001
depth^2^	−2.47 × 10^−1^	1.03 × 10^−1^	.016
T°	1.59 × 10^−1^	5.61 × 10^−1^	.777
d.eelgrass * SCL	−1.83 × 10^−4^	3.82 × 10^−5^	<.001
d.eelgrass^2^ * SCL	3.94 × 10^−7^	8.09 × 10^−8^	<.001
depth * SCL	2.12 × 10^−2^	5.70 × 10^−3^	<.001
depth^2^ * SCL	2.37 × 10^−3^	1.08 × 10^−3^	.028
T° * SCL	1.70 × 10^−2^	5.87 × 10^−3^	.004
